# Determining the Dependence of Single Nitrogen−Vacancy Center Light Extraction in Diamond Nanostructures on Emitter Positions with Finite−Difference Time−Domain Simulations

**DOI:** 10.3390/nano14010099

**Published:** 2023-12-31

**Authors:** Tianfei Zhu, Jia Zeng, Feng Wen, Hongxing Wang

**Affiliations:** 1 Key Lab for Physical Electronics and Devices of the Ministry of Education, Faculty of Electronics and Information Engineering, Xi’an Jiaotong University, Xi’an 710049, China; 18713719437@163.com (J.Z.); fengwen@mail.xjtu.edu.cn (F.W.); hxwangcn@mail.xjtu.edu.cn (H.W.); 2 Institute of Wide Bandgap Semiconductors, Xi’an Jiaotong University, Xi’an 710049, China

**Keywords:** nitrogen−vacancy center, nanocones, emission efficiency, FDTD simulations, single−crystal diamond

## Abstract

In this study, we obtained a diamond nanocone structure using the thermal annealing method, which was proposed in our previous work. Using finite–difference time–domain (FDTD) simulations, we demonstrate that the extraction efficiencies of nitrogen–vacancy (NV) center emitters in nanostructures are dependent on the geometries of the nanocone/nanopillar, emitter polarizations and axis depths. Our results show that nanocones and nanopillars have advantages in extraction from emitter dipoles with s− and p−polarizations, respectively. In our simulations, the best results of collection efficiency were achieved from the emitter in a nanocone with s−polarization (57.96%) and the emitter in a nanopillar with p−polarization (38.40%). Compared with the nanopillar, the photon extraction efficiency of the emitters in the nanocone is more sensitive to the depth and polarization angle. The coupling differences between emitters and the nanocone/nanopillar are explained by the evolution of photon propagation modes and the internal reflection effects in diamond nanostructures. Our results could have positive impacts on the design and fabrication of NV center−based micro− and nano−optics in the future.

## 1. Introduction

The optical active impurities in diamonds, also called color centers, have attracted much research interest in recent years. More than 500 different color centers exist in diamonds [1]. Many of these color centers have unique properties, including the stable fluorescence of a single color center at room temperature and the feasible control of a single, highly coherent spin associated with the color centers of diamonds [2,3]. The emission wavelengths of the known color centers of diamonds span a spectral range from ultraviolet to near infrared. Furthermore, diamonds, as a host material, have remarkable physical properties, such as a superior hardness, chemical inertness, a high transparency spectral range from ultraviolet to infrared, biocompatibility, and high thermal conductivity [4,5,6]. Hence, the color centers of diamonds can be considered as candidates for future quantum information processing applications. 

The nitrogen−vacancy (NV) center of diamonds has been identified as an excellent candidate for a wide range of applications, such as quantum computation and communication [7,8], biological detection [9,10,11,12], and high−precision quantum sensors for electromagnetic, temperature, stress, and pressure fields [13,14,15,16,17]. The NV color center has a stable photon emission performance at room temperature, and its electron spin has a long coherence time (T_2_ = 1.8 ms) [18], which is sensitive to changes in the surrounding environment and causes corresponding feedback that can be optically detected in the form of the spin state of the color center. The high sensitivity, high spatial resolution, easy initialization, manipulation, and readout, as well as the abundant energy level structure of a single NV color center render it uniquely advantageous for the field of quantum precision measurements. Atomic force microscopy uses optical or tunneling current detection methods to detect cantilever displacement caused by the force between the probe tip atom and the sample surface to obtain sample surface information; the accuracy is generally in the order of 0.1 nm. If the NV color center is implanted in the probe tip of a scanning tunneling microscope or an atomic force microscope, the spatial resolution and quantitative measurement accuracy can be greatly improved at the sub−angstrom level, and the scanning speed can also be increased. This is due to the different spatial magnetic field distributions, which cause the energy level of the NV color center to move differently. This level shift can be detected as fluorescence, which can lead to the detection of precise information, such as the surface topography of the sample [19,20]. Its highly sensitive detection is also suitable for electron or proton spin detection, and Stuadacher detected the nuclear spin signal of 104 protons in a volume of (5 nm)^3^ at room temperature using an NV color center [21]. In addition, NV color centers also have biological applications, such as the use of NV color centers for sensitive intracellular temperature detection [14] and the magnetic field detection and imaging of magnetotactic bacteria [22]. In quantum detection applications, the number of excitation photons and the detection efficiency are the main factors that determine the detection accuracy of the NV color center of the external environment [23]. At present, researching how to increase the number of exit photons is one of the main methods to increase the output signal of a single−photon source.

Single NV center emitters embedded in a piece of bulk diamond are not good for high−performance single−photon sources, as the extraction efficiency is severely limited by the lack of directionality in the NV center emission. Additionally, the total internal reflection occurring at the diamond–air interface leads to a very small critical angle (24.4°) due to the high refractive index contrast of diamonds, above which the emitter light cannot escape the bulk semiconductor [24,25]. The employment of nanophotonic structures is an elegant means to solve the total internal reflection problem, as these structures may not only improve the extraction efficiency, but also enhance spontaneous emissions in other modes [26]. Although many experimental and simulation works have researched the coupling of diamond NV color centers with nanostructures to improve the photon extraction efficiencies [27,28,29,30,31], the extraction of light from emitter dipoles in diamond nanostructures has not been considered in detail when varying the axis depth, the polarization direction of emitters and the geometry of nanostructures. A deep insight into the change law and mechanisms for the extraction efficiencies of NV color centers is important since the color centers of diamond nanostructures have various position parameters during the real formation process. In our work, we study the simulated emissions from dipoles with different positions embedded in nanocones whose size is comparable to that of a prepared sample. At the same time, we chose nanopillars with the same diameter and height as the nanocones for comparison.

We utilize the thermal annealing method to fabricate a nanocone structure. Then, combined with a nanopillar model, we simulated the NV center dipoles embedded in nanocones and nanopillars using finite−difference time−domain (FDTD) software of Lumerical Solutions 2017a, respectively. The emission performances of dipoles with different polarizations and depths in nanocones and nanopillars are studied.

## 2. Experimental and Simulations

**Figure 1:**
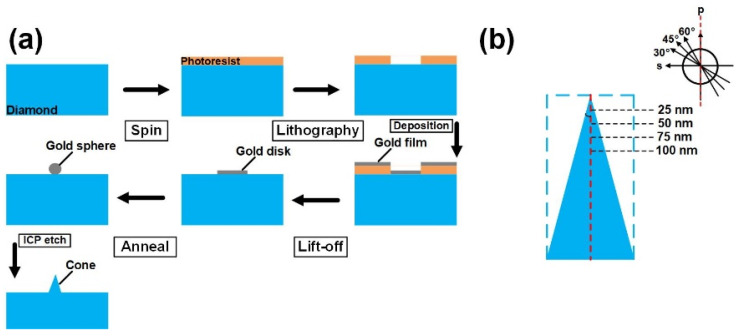
(**a**) Schematic of nanocone fabrication on diamond surface. (**b**) Diagram of modeled nanocone and nanopillar structures. Profile of nanopillar is indicated with blue dashed line. The depths and polarization angles of emitter dipoles are indicated.

In our work, we use IIa−type chemical vapor deposited (001) diamonds with a high quality for fabricating the nanostructures. The diamond samples are obtained using homogeneous growth on an HPHT diamond substrate measuring 3 mm × 3 mm × 0.5 mm with our home−built equipment. Before growth, the HPHT diamond substrate was dipped into a mixed acid of H_2_SO_4_:HNO_3_:HClO_4_ (volume ratio: 31.2:36:11.4) at 250 °C for 1 h and a mixed alkali of NH_4_OH:H_2_O_2_:H_2_O (volume ratio: 4:3:9) at 80 °C for 10 min to remove the non−diamond phase. Then, the implanted layer was epitaxially grown on an as−washed substrate. During growth, the typical growth parameters were as follows: a total gas pressure of 100 Torr, a H_2_ gas flow rate of 500 sccm, an O_2_ gas flow rate of 2.9 sccm and a CH_4_ gas flow rate of 40 sccm. The growth time was 30 h, and the thickness of the implanted layer was estimated to be 300 µm. During the growth process, the gas of O_2_ was added to restrain defect formations. After growth, the as−grown diamond was polished on the grown side, and the growth layer was cut off in the direction of the parallel growth plane. The obtained as−cut growth layer was about 250 µm. Nanostructures on the as−grown diamond surfaces were fabricated using the thermal annealing method. Figure 1a shows the fabrication flow chart. The SPR−220 photoresist (PR) was spun on the cleaned, as−grown diamond, resulting in a PR thickness of 3 µm. The standard photolithography process was used to form round hole patterns with a diameter of 1 µm. Gold disks with a thickness range of 10 nm were obtained using electron beam evaporation and lift−off processes. Then, the samples were held in a furnace for annealing at 1100 °C for 5 min. During the annealing, micro−sized gold disks turned into nanoscale−sized spheres due to the metal dewetting effect, which was used as an etching mask for the diamond nanostructure. Finally, the diamond with an optimized gold dot mask was inductively coupled plasma (ICP)−etched to obtain the nanostructure. Here, O_2_ gas was used as etching gas with a flow rate of 50 sccm. Chamber pressure and coil power were 10 mTorr and 450 W, respectively. A platen power of 25 W was applied to obtain the morphology of the nanocone. The etching time was 3 min. After ICP etching, the sample was washed in aqua regia to remove any remaining gold. The morphology of the nanostructure was characterized using scanning electron microscopy (SEM). To obtain a three−dimensional perspective, the sample was mounted at an angle of 45° to the stage.

An FDTD simulation (Lumerical Solutions Ltd, Vancouver, Canada) was used to numerically calculate the collection efficiencies of the nanostructures. In the simulation, a nanocone model of a size comparable to the sample was built. For comparison, a nanopillar model with a height and diameter equal to those of the nanocone was also modeled. A diagram of the simulated structures is shown in Figure 1b. The regular nanostructures were arranged perpendicular to the X−Y plane. In the simplified model, the refractive indexes of air and diamond were 1 and 2.42 [27], respectively. The simulation region was 2 µm × 2 µm × 2 µm. A constant−size mesh of 1 × 1 × 1 nm was used. Perfectly matched layers were employed as the boundary conditions in this simulation. In both models, electric dipoles with emission wavelengths of 637 nm were set at various depths along the central axis of the nanostructures. Dipoles with various polarization directions to the central axis were also modeled. In this work, s−and p−polarized dipoles represent NV centers with polarization orientation parallel (0°) and perpendicular (90°) to the x−y plane, respectively. The NV centers with polarization angles of 30°, 45°, and 60° to the s−orientation were also modeled, as shown in the upper−right panel of Figure 1b. A monitor was set below the nanostructure to obtain a dipole emission far−field projection [32]. The far−field projection was recalculated on the basis of Snell’s law and Fresnel equations to obtain the reflection and refraction at the interface between diamond and air. The collection efficiency can be expressed as

(1) $$\eta =\frac{I_{NA}}{I_{total}}$$

where I_NA_ is the integration of the far−field electric field intensity in the objective collection angle, and I_total_ is the source power emitted by a bulk diamond.

Our settings and calculations are equivalent to the effect that an objective with a numerical aperture (NA) of 0.95 was set for the samples to collect photons. The collecting region is represented by the area above the green dash line indicated in result part.

## 3. Results and Discussion

**Figure 2:**
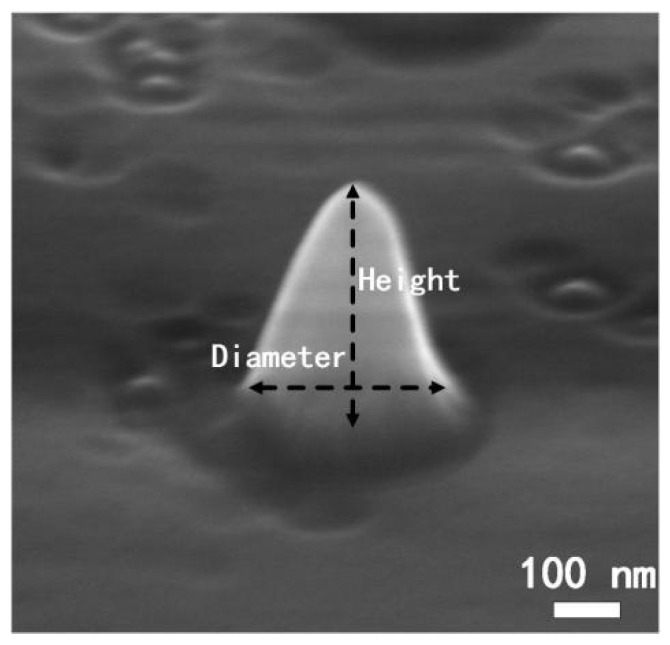
SEM images of as−fabricated diamond nanocone structure with a height of 415 nm, a base diameter of 290 nm, and a cone angle of 33.01°.

The nanostructure of the diamond surface obtained using the thermal annealing method [33] has a nanocone geometry. Achieving a nanocone structure using the thermal annealing method is advantageous since the etch mask is a gold sphere instead of a disk. The diameter of the gold sphere mask varies in height, while the disk mask does not change. Hence, diamond nanocones are easier to form with gold sphere masks than with disk masks during the etching process. With gold spheres as the etch mask, there are more possibilities to flexibly adjust the profile of the nanocone structure. Nanocones with various top angles and base diameters can be obtained by adjusting the etch parameters to control the etch selectivity of the mask and diamond. Since the thermal annealing method exhibits the advantage of large−scale preparation of nanostructures compared with conventional methods with electron beam lithography technology, it is significant to apply this as−fabricated nanocone to single−photon source preparation. Here, one typical nanocone structure is shown to study its influence on the emission efficiency of a single−photon source. The values of the diameter and visual height were 290 nm and 360 nm, respectively. Since the camera angle in SEM equipment is 70° perpendicular, the actual height is calculated to be 415 nm, with a trigonometric relationship. Additionally, the cone angle of the nanostructures is calculated to be 33.01°. From the SEM results as shown in Figure 2, the top of nanostructure is not strictly pointed.

The emitting performances of the NV center of diamond nanostructures were studied using FDTD simulations. In addition to the simulation of the nanocones, a nanopillar was also modeled for comparison. To discern the influence of (i) the nanostructure geometries, (ii) the depth of the emitter at the axis, and (iii) the polarization directions on the extraction efficiency of a NV center emitter, emitter dipoles with various polarization directions were positioned on the axis of nanocones and nanopillars at different depths. In the simulations, emitters with polarization directions of 0, 30, 45, 60, and 90° were modeled and placed in nanostructures at depths of 25, 50, 75, and 100 nm from the top, respectively.

**Figure 3:**
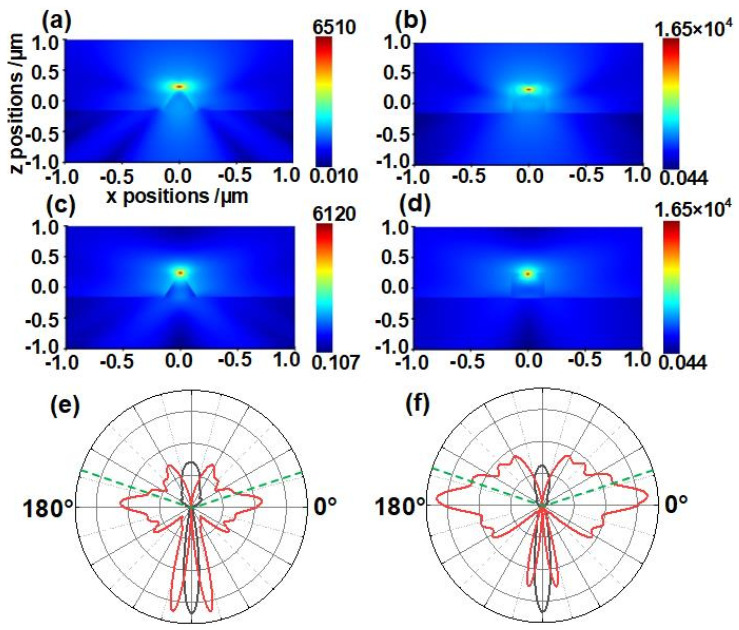
FDTD simulation results of nanocones and nanopillars. Optical field distribution images of (**a**) nanocones and (**b**) nanopillars with s−polarization emitter dipole. Optical field distribution images of (**c**) nanocones and (**d**) nanopillars with p−polarization emitter dipole. Radiation diagrams of emitters in (**e**) nanocones and (**f**) nanopillars. The black and red lines represent s− and p−polarized emitter cases, respectively. The collecting region is the area above the green dash line equivalent to the collection range of an objective with an NA of 0.95.

**Figure 4:**
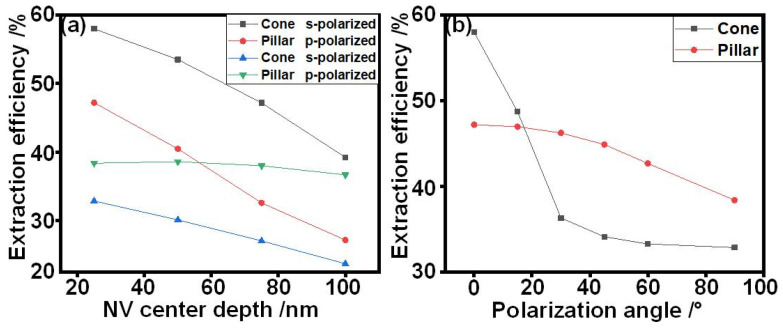
Simulated extraction efficiencies of emitters embedded in nanocones and nanopillars with various (**a**) axis depths and (**b**) polarization angles.

**Figure 5:**
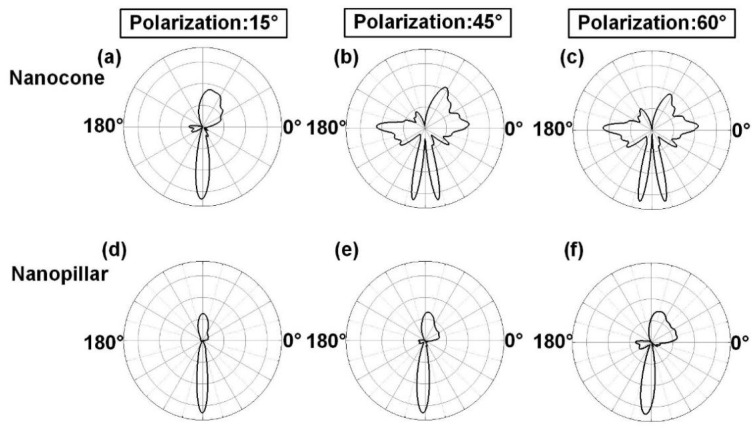
Radiation diagrams of emitters in (**a**–**c**) nanocones and (**d**–**f**) nanopillars with polarization angles of 15°, 45°, and 60°, respectively.

The simulation results are shown in Figure 3, Figure 4 and Figure 5. The optical field distributions of the nanostructures are shown in Figure 3a–d, which indicate that both the nanocone and nanopillar structures impact the emissions of the s− and p− polarized NV center emitters. The optical field near the emitter in the nanocones is slightly different from that in the nanopillars. As shown in Figure 3e,f, far−field radiation diagrams were plotted to obtain a clear view of the behavior of the photons emitted from the emitters. Both in the nanocones and nanopillars, the photons emitted from s−polarized emitter dipoles are confined mainly along the axis of nanostructures, while those from the p−polarized emitter dipoles propagate in all directions. More photons propagate along the axis direction, leading to a higher extraction efficiency due to an increase in photons in the collect region of lens upward in the sample. We calculated the extraction efficiency of s−polarized emitters in the nanocones and nanopillars to be 57.96% and 47.20%, respectively. Two effects of the nanocone top may result in a higher efficiency in the nanocones than that in the nanopillars: (i) the reflection was minimized by the top facet of the guided mode back to the diamond; (ii) collimation was made easier, which facilitates photon collection with external optics of a limited aperture [24,26]. However, the extraction efficiency of p−polarized emitters in the nanocones is 32.89%, which is lower than the value of 38.40% in the nanopillar case. This may be due to the fact that the photons emitted from p−polarized emitters that propagate in the radiative mode in the nanocone reflect downward at sidewall interfaces with a higher probability than those in the nanopillars.

In polycrystal nanostructures, the polarization angles of the emitters to the surface are varied since the orientations of crystal particles in polycrystal diamonds are random. Even in single−crystal diamonds, emitters with various polarizations could be formed using ion implantation techniques in actual fabrication. Meanwhile, the depths of the NV centers could be different during ranging, although the energies of implantation ions are the same. This could cause the color centers prepared in one batch to have different positions. Hence, the influence of the emitter’s polarization and depth on the extraction efficiencies is important in the fabrication and study of NV centers. Since it is still difficult to experimentally confirm the polarization angle of a single−photon source, we used the FDTD software to simulate the photon emission properties of different polarization angle emitters in nanocones and nanopillars. 

Figure 4 shows the simulation results for the dependence of emitter extraction efficiencies on the emitter’s positions. The photon extraction efficiencies of s− and p−polarized emitters positioned in nanocones and nanopillars at various depths are shown in Figure 4a. Both in the nanocones and nanopillars, the extraction efficiencies of the s−polarized emitters decrease with an increased depth of the emitter. However, the efficiencies of the p−polarized emitters in the nanocones and nanopillars are different. The efficiency decreases with the depth of the emitter in the nanocones, while that remains almost unchanging in the nanopillars.

The extraction efficiencies of the emitters with various polarizations in the nanocones and nanopillars at a depth of 25 nm from the top are shown in Figure 4b. The efficiencies of the nanocones decrease with an increase in the polarized angle. When the angle is less than 30°, the efficiency experiences a dramatic decrease. When the angle is greater than 30°, the efficiency decreases slowly and remains unchanging. In the case of the nanopillars, the efficiencies remain unchanging when the polarization angle is less than 30°, which is lower than that achieved in the nanocones. With further increases in the polarization angle, the efficiency decreases slowly, which is higher than that achieved in the nanocones.

Our results demonstrate that the dependence of photon extraction efficiency in the nanocone on the depth, and polarization angle is different from that in the nanopillar. For the s−polarized emitters, photons are mainly transmitted in the guided mode in the nanocone and nanopillar, in which the emission efficiency of emitters in these nanostructures decreases with the increase in depth as the distance the photon travels become longer. For the p−polarized emitters, the nanocone structure has lower photon extraction efficiency compared with that of the nanopillars. From Figure 3e,f, the polar plots show that the photons emitted from p−polarized dipoles mainly propagate in radiative modes in the nanostructures. The radiative amplitude of an emitter in nanocones is narrower than that in the nanopillars, which may result in the lower extraction efficiency of the nanocones. In comparison with the nanopillars, the extraction efficiency of the nanocones is more sensitive to the increase in emitter depth since the diameter of the nanocones at the emitter depth increases with the emitter depth. Hence, with the increase in the emitter depth, the distance between the emitter and side wall increases, leading to a decrease in the photon extraction efficiency of the nanocones. Meanwhile, the slope sidewall in the nanocone may reflect more emitted photons back into the diamond body at the interfaces, which would decrease photon extraction in the nanocones in comparison with that of the nanopillars.

The preference in emission coupling to a desired optical mode within a photonic structure can be expressed as the β−factor [24,34].

(2) $$\beta =\frac{\Gamma }{\Gamma +\gamma }$$

where Γ is the spontaneous emission rate to the desired mode supported by the nanostructure, and γ is the spontaneous emission rate in all the other modes. The extraction efficiency depends on the outcoupling efficiency of photons in the desired mode and the directionality of the outcoupled light field. For the 0° polarization dipole (s−polarized), the guided mode is the desired mode for photon outcoupling. With the increased polarization angle, Γ decreases, while γ increases since the guided mode rate is decreased. Figure 5 depicts radiation diagrams under the polarization states from s− to p−polarizations with polarization angles of 15°, 45°, and 60°, respectively. With the increase in the polarization angle, the photon transmission mode in the nanocone changes more quickly from the guided mode to the radiation mode than that of the nanopillar. This means that the β−factor in the nanocone decreases faster than that in the nanopillar. This difference between the nanocones and nanopillars may be attributed to their sidewall angles, which affect the reflection of emitted photons and the directionality of outcoupled light fields.

Based on the above, the extraction efficiency of the nanocone structure fabricated using the thermal annealing method is superior to that in the nanopillars with s−polarized emitters, and it is sensitive to the depth and polarization angle of the emitters. For the p−polarized emitters, the nanopillar structure may be the best choice to improve the extraction efficiency. These results are similar to those published by Nicklas [35]. In Nicklas’ work, the s−polarized emitters in the nanocones and nanopillars exhibit higher photon extraction efficiencies compared with those in the p−polarization cases. The photon extraction efficiencies of the p−polarized emitter in the nanopillar is higher than that in the nanocone. Although they performed electromagnetic modeling of the dipole emitter’s emission behavior using Maxwell’s equations and used different material parameters of diamonds, the influence of the positions of the emitters in the nanostructures on the emission efficiencies exhibited a similarity. In both works, the study of the polarization of emitters in nanocones and nanopillars is focused on the relationship between the polarization angle of the emitter and sidewalls. Our results demonstrate that the emission mode changed from the guided mode to the radiative mode, with the polarization changing from s−polarization to p−polarization. Combined with the sidewall angle change, the emissions in the nanocone and nanopillar exhibit different results.

In general, the emission properties of an NV center of a diamond exhibit a complicated dependence on the geometry of nanostructures, the depth, and the polarization of the emitters. Due to this complicated dependence, we believe that our simulation results are valuable in providing guidance on suitable designs and accurate analysis for the fabrication and application of nanostructure−coupled emitters and developing optics and photonics to become mainstream disciplines. Combined with the advantages of our developed thermal annealing method, it has industrial production of single−photon sources with a high photon extraction efficiency. Furthermore, our results also apply to other single−photon sources of color centers and have significance for the application of the other color centers of diamonds and in other materials.

## 4. Conclusions

In this study, a typical diamond nanocone obtained using the thermal annealing method was characterized and used as a simulated prototype. The optical simulation of the emission properties of NV center emitters based on the as−fabricated diamond nanocone structure model was carried out, and a model of a nanopillar of a comparable size to a nanocone was also built. The extraction efficiency of the emitter is dependent on the geometry of the nanostructures, depth, and polarization of the emitter. For s−polarization emitters, the extraction efficiencies of NV centers of both the nanocones and nanopillars decrease with an increase in the emitter depth. For the p−polarized case, the extraction efficiencies of the NV center of nanocones decrease with an increase in depth, while those of the nanopillars remain unchanged. With various polarization angles, propagation−mode photons in the nanocones change from the guided mode to the others, highlighting a sensitive efficiency response to the angles. In the case of the nanopillars, most of the guided mode is maintained even after the angle reaches 60°; as a result, the efficiencies remained almost unchanged when the angle was less than 60°. The best efficiency of 57.96% was obtained from the emitters in the nanocones with s−polarization dipoles, while an efficiency of 38.40% was obtained from the emitters in the nanopillars with p−polarization dipoles. In the future, our results could assist with the design and fabrication of high−performance nano−optics.

## Data Availability

The data presented in this study are available on request from the corresponding author.
